# Lipid Biomarkers in Glioma: Unveiling Molecular Heterogeneity Through Tissue and Plasma Profiling

**DOI:** 10.3390/ijms26199820

**Published:** 2025-10-09

**Authors:** Khairunnisa Abdul Rashid, Norlisah Ramli, Kamariah Ibrahim, Vairavan Narayanan, Jeannie Hsiu Ding Wong

**Affiliations:** 1 Department of Biomedical Imaging, Faculty of Medicine, Universiti Malaya, Kuala Lumpur 50603, Malaysia; khairunnisa_rashid@um.edu.my (K.A.R.); norlisahramli@gmail.com (N.R.); 2 University of Malaya Research Imaging Centre, Faculty of Medicine, Universiti Malaya, Kuala Lumpur 50603, Malaysia; 3 Department of Biomedical Science, Faculty of Medicine, Universiti Malaya, Kuala Lumpur 50603, Malaysia; kamariahibrahim2106@um.edu.my; 4 Division of Neurosurgery, Department of Surgery, Faculty of Medicine, Universiti Malaya, Kuala Lumpur 50603, Malaysia; nvairavan@um.edu.my

**Keywords:** glioma, lipid, lipid biomarkers, metabolic dysregulation, liquid chromatography-mass spectrometry

## Abstract

Gliomas are aggressive brain tumours with diverse histological and molecular features, complicating accurate diagnosis and treatment. Dysregulated lipid metabolism contributes to glioma progression, and analysing lipid profiles in plasma and tissue may enhance diagnostic and prognostic accuracy. This study investigated lipid dysregulation to identify key lipid signatures that distinguish glioma from other brain diseases and examined the associations between lipid biomarkers in glioma tissue and plasma. Biospecimens from 11 controls and 72 glioma patients of varying grades underwent lipidomic profiling using liquid chromatography-mass spectrometry. Univariate and multivariate analyses identified differentially abundant lipids, and correlation analysis evaluated the associations between tissue and plasma biomarkers. Lipidomic analysis revealed distinct lipid profiles in the tissues and plasma of glioma patients compared to those of controls. Prominent lipid metabolites in glioma tissues included LPC 21:3 (AUC = 0.925), DG 43:11 (AUC = 0.906), and PC 33:1 (AUC = 0.892), which served as effective biomarkers. Conversely, in plasma, lipid metabolites such as phosphatidylethanolamine (PE 21:3, AUC = 0.862), ceramide-1-phosphate (CerP 26:1, AUC = 0.861), and sphingomyelin (SM 24:3, AUC = 0.858) were identified as the most promising lipid biomarkers. Significant positive and negative correlations were observed between the tissue and plasma lipid biomarkers of glioma patients. Lipidomic profiling revealed aberrant lipid classes and pathways in glioma tissues and plasma, enhancing understanding of glioma heterogeneity and potential clinical applications.

## 1. Introduction

Gliomas are highly aggressive and heterogeneous brain cancers, often diagnosed late due to non-specific symptoms like headaches, dizziness, vomiting, personality changes, or neurological deficits [1]. Magnetic resonance imaging (MRI) is the primary imaging modality for suspected brain tumours but definitive diagnosis requires histological examination of biopsy or resection samples [2]. Unfortunately, diagnosis often comes too late for effective treatment, with a high likelihood of recurrence post-therapy [3]. The variable clinical manifestations and lack of reliable screening tools further complicate early glioma detection. Thus, developing accurate, rapid, and effective diagnostic techniques is critical for improving the prognosis of glioma patients [4].

Metabolic alterations, particularly in lipid metabolism, are hallmarks of cancer, driving tumour growth and influencing therapeutic response [5]. Lipids and their intermediates are heterogeneous molecules essential for brain structure and function, constituting about 50% of the brain’s dry weight [6]. Lipidomic is the quantitative measurement of lipids in cells, tissues, body fluids, or organisms over a period of time [7]. A lipidomic analysis will reveal a lipid profile, which encloses information on the composition and abundance of individual lipids derived from the initial materials, providing insights into the molecular mechanisms of lipid metabolism abnormalities [8].

Gliomas exhibit significant lipid changes, with higher lipid content in tumour tissues compared to normal tissues [9]. Enhanced fatty acid metabolism and fatty acid synthase (FAS) expression, were reported in glioma, support tumour energy demands suggesting lipid metabolites as potential biomarkers reflecting disease mechanisms [10,11,12]. Glycerolipid metabolism is often dysregulated in gliomas, with elevated DG linked to malignant transformation and TGs consumed by GBM for energy production. [13] A high level of phospholipids, especially phosphatidylcholine, was also detected in glioma compared to the normal tissues [14]. Dysregulation of sphingolipid metabolism, with increased sphingomyelin (SM) was reported in HGG, and elevated S1P levels contribute to treatment resistance and promote cell growth, division, and blood vessel formation [15]. GBM tissue was found to produce high levels of cholesterol to maintain its growth and development [16]. Increased level of 1-oleyl cholesterol and tetrahydrocorticosterone, in the grade III gliomas [13]. Identifying lipid metabolites can lead to the discovery of biomarkers that reflect the biological mechanisms and pathogenic states of the disease.

Furthermore, the development of cancer screening technologies based on peripheral blood testing, including liquid biopsy, has received more attention in recent years [17]. Cancer cells secrete various components, including lipids, which may indicate cellular metabolic and genetic changes, making their profiling in biofluids attractive, especially in early detection phases [18]. Circulating lipid metabolites are implicated in physiological and pathological mechanisms [19]. Several studies have shown distinct plasma lipidomes between brain disease and healthy states [20,21]. Increased plasma ceramide levels have been associated with tumour progression and malignancy, particularly in higher-grade gliomas [22]. Conversely, reduced plasma glycerophospholipid levels have also been reported in glioma. Nevertheless, studies examining lipid alterations in brain cancer, particularly glioma, remain limited.

In a prior systematic review, diverse lipid alterations with carcinogenic and anticarcinogenic roles in glioma [23]. Building on this knowledge, the present study aimed to investigate lipidomic changes in tissues and plasma from glioma patients and control subjects. Liquid chromatography–electrospray ionisation quadrupole time-of-flight mass spectrometry (LC/ESI-Q-TOF MS) was employed for untargeted lipidomic analysis. Differentially expressed lipid metabolites were identified through bioinformatics analyses to screen potential biomarkers for glioma.

## 2. Results

### 2.1. Patient Characteristics

This study included 83 glioma patients, comprising 29 with low-grade glioma (LGG) and 54 with high-grade glioma (HGG). LC-MS-based plasma lipidomics was conducted on 11 controls and 72 glioma samples (26 LGG, 46 HGG), while untargeted tissue lipidomics included 8 controls, 14 LGG, and 31 HGG specimens.

Table 1 summarises demographic and clinical features. LGG predominated in females (60.7%) and younger adults (mean age: 37.6 years), while HGG was slightly more frequent in males (51.9%) and older adults (mean age: 46.0 years). Chinese ethnicity was the most represented in both groups. LGG patients had a normal BMI (mean: 23.2 kg/m^2^), whereas HGG patients were overweight (mean: 25.7 kg/m^2^). Seizures were the most common symptom in LGG, while HGG often presented with multiple symptoms, including seizures and headaches.

Tumours were primarily located in the frontal and temporal lobes. Histological grades were distributed as follows: Grade I (*n* = 4), Grade II (*n* = 23), Grade III (*n* = 15), and Grade IV (*n* = 39). Glioblastomas accounted for 79.1% of cases. Control samples included non-glioma diagnoses such as mesial temporal sclerosis (36.4%) and dysplasia (18.2%). Challenges in sample collection, including the COVID-19 pandemic, contributed to the diversity of the control group.

Treatment varied between groups, with most LGG patients undergoing surgery alone (75%), while HGG patients required surgery with chemotherapy and/or radiotherapy (85.2%). Maximum safe resection was performed in 71.4% of LGG and 68.5% of HGG cases.

LGG patients had higher Karnofsky Performance Scale (KPS) scores (median: 80) compared to HGG patients (median: 60). LGG showed longer overall survival (median OS: 25.9 months) and time-to-progression (median TTP: 13.1 months) than HGG (median OS: 15.4 months; median TTP: 3.4 months).

### 2.2. Lipidomic Differentiation of Gliomas with Control Group

**Figure 1:**
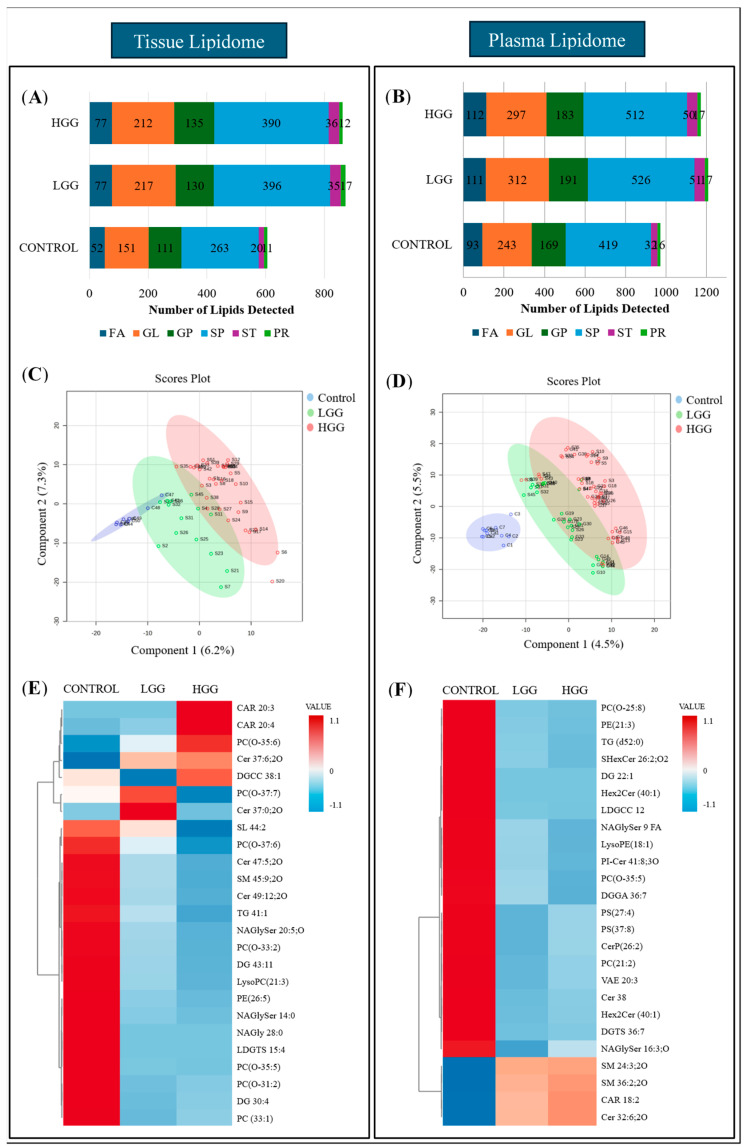
Comparison of plasma and tissue lipidomic profiles between glioma and control subjects. (**A**) Relative quantification of lipids in tissue. (**B**) Relative quantification of lipids in plasma. (**C**) Supervised clustering analysis, PLSDA visualised the separation between control and glioma groups based on lipid composition in tissue. (**D**) PLSDA analysis illustrates the separation of control and glioma subgroups according to the lipid composition in plasma. Sample groupings are displayed with different colours: control in blue, LGG in green, and HGG in red. (**E**) Clustering heatmap of lipid composition in tissue. (**F**) Clustering heatmap of lipid composition in plasma. The heatmaps were arranged as the top 25 lipids from the control and glioma groups were included and ranked based on *t*-tests to highlight the most distinct patterns. The colour bar indicates the abundance of the lipid species (red shows the molecules with increased levels in particular samples, while blue reflects decreases in lipid species).

Lipidome analysis revealed increased lipid composition in glioma tissues compared to controls (Figure 1A). LGG and HGG tissues contained 872 and 862 unique lipid species, respectively, across six categories (FA, GL, GP, SP, ST, PR), while controls had 608 species. Plasma analysis revealed 1171 and 1208 lipid species in HGG and LGG, respectively, compared to 962 in controls (Figure 1B). Sphingolipids and glycerolipids were the predominant categories, with elevated levels in glioma tissues and plasma.

Univariate and multivariate analyses characterised lipid expression differences. Tissue lipid analysis identified 18 differentially expressed lipid species, comprising 15 downregulated and three upregulated lipids. Plasma analysis identified 47 distinct lipids (*p* < 0.05), with 89% downregulated, primarily comprising sphingolipids (34%), glycerolipids (29.8%), and glycerophospholipids (21.3%). Five lipids from fatty acyl and sphingolipid families showed increased levels in glioma samples (Supplementary Table S1).

PLS-DA visualised lipid clustering, showing partial overlap between control and LGG tissue but distinct separation from HGG (Figure 1C). Plasma samples showed a clear separation between the control and glioma groups (Figure 1D). Heatmap analysis highlighted increased levels of carnitine, phosphocholine, and ceramide in tumour tissue, accompanied by decreased sulfunolipid, sphingomyelin, triacylglycerol, and fatty acyls (Figure 1E). Plasma lipidome analysis revealed reduced glycerolipids and glycerophospholipid derivatives, including lysophosphatidylethanolamine, phosphatidylserine, and phosphatidylcholine, while fatty acyl and sphingomyelin levels were elevated (Figure 1F).

### 2.3. Predictive Performance of Lipid Biomarkers

The diagnostic accuracy of differential lipids for distinguishing glioma from controls was assessed using ROC curves, measuring the area under the curve (AUC) as an indicator of diagnostic performance. Lipids with an AUC > 0.75 demonstrated strong diagnostic potential. Six tissue lipids and ten plasma lipids showed high diagnostic value (Table 2, Supplementary Figures S1 and S2). Tissue biomarkers, all significantly reduced in gliomas, included Cer 39:5 (AUC = 0.986), LPC 21:3 (AUC = 0.928), and DG 43:11 (AUC = 0.911). Multivariate ROC analysis confirmed good discrimination (AUC = 0.927).

**Figure 2:**
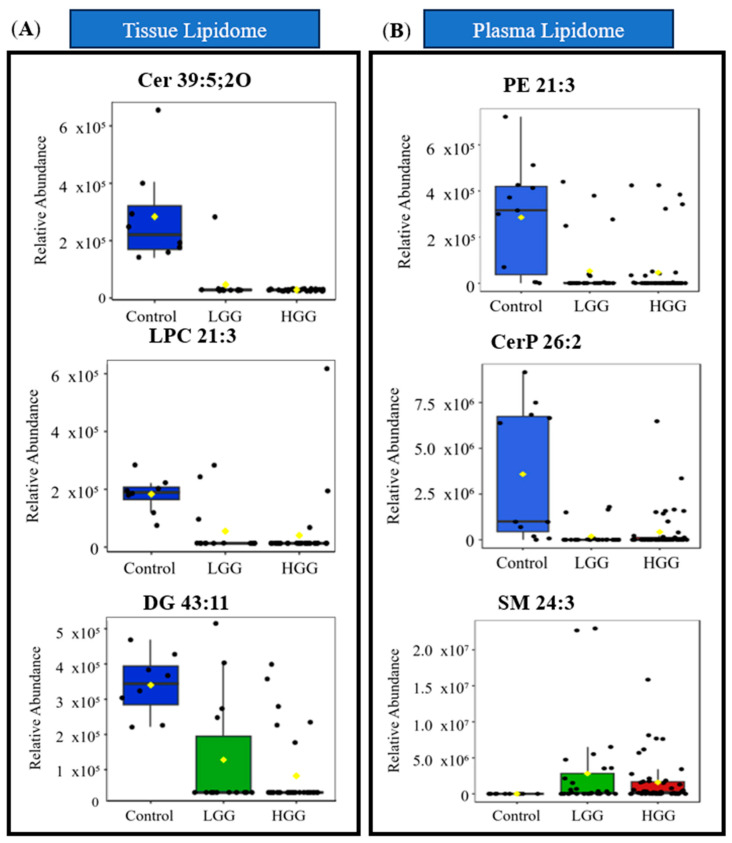
Distribution of relative abundance of significant lipid biomarkers (**A**) in tissue and (**B**) plasma. The green box indicates lipids in tissue, while the blue box represents lipids from plasma. Sample groupings are displayed with different colours: Control in blue, LGG in green and HGG in red. Abbreviations: Cer ceramide, CerP ceramide 1-phosphate, DG diacylglycerol, DGGA diacylglyceryl glucuronide, LysoPC lysophosphocholine, NAGlySer N-acylglycine serine, PC O ether-linked phosphatidylcholine, PE phosphatidylethanolamine, PI-Cer ceramide phosphoinositol, SM sphingomyelin.

In plasma, PE 21:3 (AUC = 0.862), CerP 12:1;2O/14:1 (AUC = 0.861), and SM 15:3;2O/9: (AUC = 0.858) exhibited strong diagnostic performance, with multivariate ROC yielding an AUC of 0.932. Boxplots in Figure 2 show the relative abundance of lipid biomarkers in glioma patients.

Overall, six tissues and ten plasma lipids were identified as potential biomarkers, all of which were significantly downregulated in glioma tissue. These findings highlight their utility in differentiating glioma from non-gliomatous diagnoses.

### 2.4. Altered Lipids Metabolites in Plasma and Associated with Metabolic Pathways

**Figure 3:**
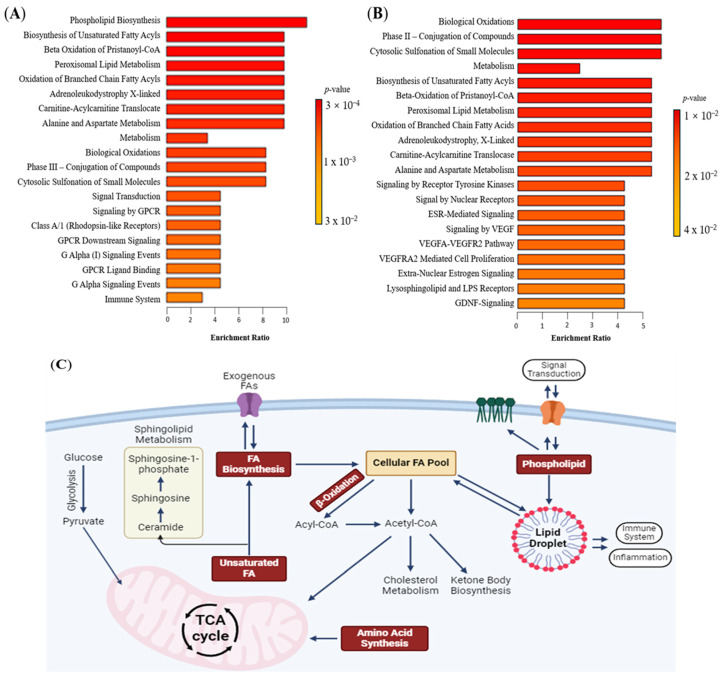
Lipidomics schema of significantly altered lipids and pathways in plasma between glioma and control. (**A**) Pathway enrichment in tissue between control and glioma. (**B**) Pathway enrichment in plasma between control and glioma. From yellow to red, the *p*-value is from large to small, indicating that the degree of enrichment is becoming more and more significant. There are lipid pathways on the vertical axis and enrichment factors on the horizontal axis, the higher the value, the greater the degree of enrichment. (**C**) Schematic figure of dysregulated lipid pathway in glioma. The red box represents the dysregulated pathway. Abbreviations: Acetyl CoA acetyl coenzyme A, ESR oestrogen, FA fatty acyl, GDNF glial cell line-derived neurotrophic factor, GPCR G-protein-coupled receptor, TCA tricarboxylic acid, VEGF vascular endothelial growth factor, VEGFA vascular endothelial growth factor—A level, VEGFR vascular endothelial growth factor receptor.

Enrichment analysis identifies alterations in metabolic pathways associated with cancer. In both tissue and plasma, similar lipid metabolism pathways associated with glioma were found, including fatty acyl biosynthesis, beta-oxidation, sphingolipid metabolism, and carnitine metabolism (Figure 3A,B). Glioma tissues also exhibited enrichment in peroxisomal lipid metabolism and phase II biotransformation processes. In glioma plasma, lipid pathways linked to cell signalling, such as receptor tyrosine kinase (RTK), oestrogen (ESR) signalling, vascular endothelial growth factor (VEGF), glial cell-derived neurotrophic factor (GDNF), and G-protein-coupled receptor (GPCR), were enriched. These pathways contribute to neuroinflammation, angiogenesis, and immune response, supporting cancer progression [24,25]. Figure 3C illustrates the connections between these enriched lipid pathways, with fatty acyls as central metabolites.

### 2.5. Correlation Between Lipid Biomarkers in Plasma and Tissue

**Figure 4:**
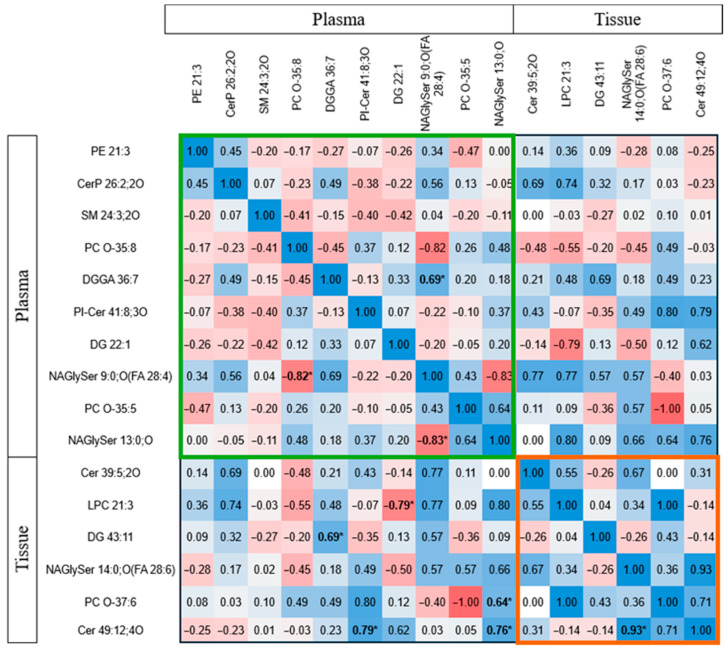
Spearman correlation (r) between lipid biomarkers in plasma and tissue in glioma patients. The colour code depicts the correlation strength, with blue representing positive correlation and red representing negative correlation. The bolded * indicates the significantly correlated parameters. The green box represents the correlations between plasma lipid biomarkers, whereas the orange box includes the correlation between tissue lipid biomarkers. Abbreviations: Cer ceramide, CerP ceramide 1-phosphate, DG diacylglycerol, DGGA diacylglyceryl glucuronide, LPC lysophosphatidylcholine, NAGlySer N-acylglycine serine, PC O ether-linked phosphotidylcholine, PE phosphatidylethanolamine, PI-Cer ceramide phosphoinositol, SM sphingomyelin.

Studies explored lipid correlations between tissue and plasma in glioma patients. Figure 4 shows Spearman correlation values, highlighting notable negative correlations in plasma, such as between fatty acyl N-acylglycine and phosphocholine species (r = −0.82, *p* = 0.002), and between N-acylglycine derivatives (r = −0.83, *p* = 0.011). N-acylglycine levels positively correlated with glycerolipids. In tissues, N-acylglycine correlated with long-chain ceramide (r = 0.93, *p* = 0.001). Additionally, simple lipids like diacylglycerol and ceramide in tissues correlated with complex lipids in plasma. For instance, ceramide (Cer 49:12) correlated with plasma ceramide phosphoinositol (r = 0.79, *p* = 0.021), and diacylglycerol correlated with plasma diacylglycerol glucuronide (r = 0.69, *p* = 0.019). Phosphocholine (PC(O-37:6)) and ceramide in tissue also correlated with fatty acyl N-acylglycine in plasma. Conversely, lysophosphocholine (LPC 21:3) in tissue negatively correlated with diacylglycerol (DG 22:1) in plasma (r = −0.786, *p* = 0.021). These correlations suggest that lipids in glioma tissues and plasma are interconnected.

## 3. Discussion

The high incidence and mortality of brain tumours highlight the need for effective diagnostic methods. Current diagnostics, such as imaging and histology, have limitations. Conventional MRI aids in surgical planning but cannot reliably differentiate advanced tumours and is prone to variability [26]. Understanding glioma biology is key to improving diagnosis, treatment, and prognosis. Molecular phenotyping is crucial for identifying abnormalities in cancer tissue [27]. Lipid biomarkers have been utilised in various cancers, including breast, lung, prostate, and colorectal [28,29]. Developing new diagnostic and intervention strategies is essential for glioma, with tumour- and fluid-based biomarkers offering more timely and comprehensive diagnosis and furthering research into glioma pathophysiology [30].

### 3.1. Tissue-Derived Lipid Biomarkers and Dysregulated Lipid Pathway

This study applied untargeted lipidomics to identify lipid biomarkers and dysregulated pathways in glioma tissues and plasma. UPLC-QTOF-ESI+ MS results, supported by statistical models, revealed six potential lipid biomarkers in glioma tissue. Key biomarkers included LPC 21:3 (AUC = 0.925), DG 43:11 (AUC = 0.906), and PC 33:1 (AUC = 0.892). PLSDA analysis revealed significant lipid heterogeneity between tumour and control groups, indicating distinct lipid profiles associated with abnormal metabolic pathways. In low-grade cancers, the differences in gene expression or other molecular profiles between the cancer and control samples may not be as pronounced as in advanced stages, leading to more overlap. Similar trends have also been reported in tumour-adjacent tissues (NAT) from several cancers, including bladder and uterus, where differential gene expression profiles exhibit partial overlap with those of normal tissues [31]. Additionally, several metabolites have been found to overlap between individuals with early-diagnosed breast cancer and healthy controls [32].

The lipid panel (LPC 21:3, PC 33:1, DG 43:11, PC-O 37:6, Cer 37:6) effectively distinguished tumour tissues from controls. Phospholipids, including LPC and PC-O, play key roles in biological processes [33]. Studies have shown that phosphatidylcholine (PC) levels are altered in cancer tissues, with specific changes observed in tumour tissues compared to normal tissues. For instance, in hepatocellular carcinoma (HCC), several polyunsaturated PC species such as PC34:2, PC36:3, and PC40:7 were reported to be decreased in tumour tissues. At the same time, lipidomic study revealed elevated levels of phospholipids, including PC, in cancer tissues compared to normal mucosa [34,35]. Additionally, research indicates that lysophosphatidylcholine (LPC) levels are often decreased in various cancer tissues, suggesting a potential role in tumour biology and as a biomarker. Studies have shown a reduction in LPC (16:0) levels in gastric cancer tissues, where it is reported that this is associated with overexpression of lysophosphatidylcholine acyltransferase 1 (LPCAT1), an enzyme that converts LPC to PC, indicating a shift in lipid metabolism within tumour cells [36]. Lipidomic analyses revealed that LPC species, such as LPC (16:0) and LPC (18:2), were nearly absent in tumour regions compared to adjacent healthy tissue, indicating significant lipid alterations in cancerous tissues [37].

A similar observation has been reported in human colon cancer, where levels of diacylglycerol (DG), particularly 1,2-sn-diacylglycerol, are significantly reduced compared to normal tissue. This reduction is evident in tumours regardless of the presence or absence of c-K-ras mutations [38]. DG, a key mediator in lipid metabolism and cell signalling, showed reduced levels in glioma tissues, likely due to its conversion to fatty acids (FA) for energy. DG also influences cancer progression via protein kinase C signalling [39]. Sphingolipid metabolism, which is disrupted in gliomas, plays a crucial role in cancer. Ceramide, a tumour-suppressor lipid, is elevated in glioma tissues and linked to malignant progression and drug resistance. Studies have shown that ceramide levels are decreased in certain cancer tissues, where in human glioma specimens, ceramide content was found to be five times lower compared to normal grey matter [40]. In contrast, sphingosine-1-phosphate (S1P) levels were nine times higher [41]. In human glioma specimens, ceramide content was found to be five times lower compared to normal grey matter, while sphingosine-1-phosphate (S1P) levels were nine times higher [42]. These findings suggest that decreased ceramide levels may play a role in tumour progression and could be a potential target for therapeutic intervention.

### 3.2. Plasma-Derived Lipid Biomarkers and Dysregulated Lipid Pathways

Plasma lipidomic analysis identified biomarkers differentiating glioma from non-glioma patients, including NAGlySer 9:0, O (FA 28:4), DG 22:1, DGGA 36:7, Cer 14:6, CerP 36:2, PI-Cer 41:8, SM 36:2, PS 37:8, LPE 18:1(d7), and PC O-17:8. The PLSDA model confirmed distinct separation between glioma and control groups, indicating the prognostic value of fatty acyls, sphingolipids, and phospholipids. Lipid leakage into plasma, influenced by lipid solubility and compromised blood–brain barrier (BBB) integrity in gliomas, could explain these variations [43].

Fatty acyl metabolism deregulation is linked to tumour staging and malignancy. Fatty acyls contribute to carcinogenesis through immune responses and cellular signalling [44]. Low short-chain acylcarnitine levels in preoperative plasma were associated with aggressive disease. Reduced glycerolipids like diacylglycerol may reflect increased energy demands or decreased lipase production [45]. Sphingolipids, including ceramide and sphingomyelin, regulate key processes in glioma, with elevated ceramide levels linked to tumour progression [46]. Our findings align with previous studies reporting elevated plasma sphingomyelin levels in ALS patients, including increased SM(d18:1/16:0) [47,48]. Furthermore, sphingomyelin phosphodiesterase 1 (SMPD1), overexpressed in glioblastoma, catalyses ceramide production, indicating active tumour growth [49].

Decreased glycerophospholipid levels, observed in many cancers, suggest a shift in metabolites to tumour tissue to support proliferation. Similar findings have also been reported in Alzheimer’s disease, where changes in serum LPC levels were associated with β-amyloid and phosphotau pathology [50]. Plasma LPC, which facilitates the transport of PUFAs such as DHA across the blood–brain barrier via the MFSD2A transporter, has been linked to a lower risk of cognitive decline and dementia in observational studies. Consequently, reduced LPC levels may hinder PUFA delivery to the brain, potentially increasing susceptibility to neurodegenerative processes [51]. Higher PC turnover and disrupted PS distribution, linked to apoptosis and clotting, further indicate tumour progression [52]. Additionally, lysophosphatidylethanolamine levels are inversely correlated with glioma progression [53].

### 3.3. Correspondence of Biomarkers Between Tissue and Plasma

Healthy and cancerous plasma and tissue have distinct lipid profiles. This study explored associations between preoperative plasma biomarkers in glioma patients, revealing potential pathophysiological connections. Simple lipids in tissue correlated with complex lipids in plasma, such as diacylglyceride in tissue and diacylglyceryl glucuronide in plasma. Ceramide levels in tumour tissue also correlated with complex sphingolipid species in the plasma [54].

The mechanisms by which metabolites exit the tumour and enter the bloodstream are complex and not fully understood. One theory suggests lipid compounds are transported via exosomes, reflecting primary tumour characteristics [55]. Extracellular vesicles secreted by cancer cells can alter adjacent stromal cells. Tumours disrupt BBB integrity, leading to heterogeneous vasculature, non-uniform permeability, and molecule efflux, complicating biomarker transport [56].

Despite the significant contributions of this study, several limitations should be acknowledged. Firstly, the study was constrained by a relatively small sample size, which may have affected the generalisability and robustness of the identified lipid biomarkers. A larger cohort is required to validate these findings and confirm that the lipidomic signatures observed are consistent across diverse patient populations. Additionally, the study focused primarily on specific lipid profiles and did not extensively explore external factors such as lifestyle and medications that might influence lipid metabolism. This narrow focus may limit the understanding of the full metabolic landscape associated with gliomas. Future research should address these issues to improve early glioma diagnosis and patient management.

## 4. Materials and Methods

### 4.1. Study Population

**Figure 5:**
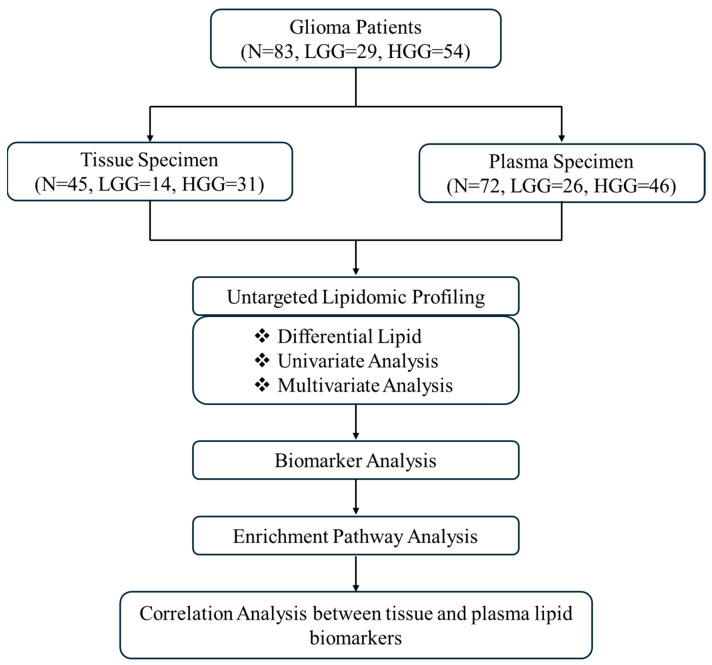
Flowchart of this study. Abbreviations: HGG high-grade glioma, LGG low-grade glioma, *N* number of samples.

Subjects were recruited from the Neurosurgical Clinic, University of Malaya Medical Centre, Malaysia, between November 2016 and November 2022. Ethical approval (MECID No: 2019823-7773) and written informed consent were obtained. The research workflow is illustrated in Figure 5. The study included 83 patients and 11 control subjects aged between 18 and 78 years.

Inclusion criteria:

1. Histologically confirmed glioma (Grade I to IV) based on World Health Organisation (WHO) classification of central nervous system tumours (4th Ed). Grades I and II are subclassified as low-grade glioma (LGG), while Grades III and IV are subclassified as high-grade glioma (HGG).
2. Participants aged 18 years or older at the time of diagnosis.

Exclusion criteria:

1. Patients with benign non-tumorous lesions were excluded from the study.
2. Cases involving metastatic brain tumours were not included.
3. Individuals with a prior history of central nervous system (CNS) infection or head trauma were excluded.
4. Paediatric cases were not considered in this study.

Clinical information and demographic data of the patients were obtained from hospital electronic medical records.

### 4.2. Glioma Biospecimens Collections

Glioma tissue samples were obtained from patients undergoing tumour resection. Control tissues came from craniotomy patients with non-malignant conditions (e.g., epilepsy surgery). Blood samples were collected after overnight fasting and centrifuged to separate plasma, stored at −80 °C. Brain tissues were snap-frozen in liquid nitrogen.

### 4.3. Lipid Extraction from Tissues

Frozen tissues were thawed on ice, lyophilised, and homogenised with PBS/MiliQ water (1:10) using a bead homogeniser. A 10 μL homogenate aliquot was mixed with chilled chloroform/methanol (180 μL, 1:2 *v*/*v*), vortexed, and incubated at 4 °C for 1 h. Additional chloroform (60 μL) and water (50 μL) were added, followed by vortexing and centrifugation (10,000 rpm, 7 min). The organic phase was collected and re-extracted with 500 μL chloroform. Combined extracts were concentrated in a vacuum concentrator, reconstituted in 100 μL chloroform/methanol (1:1 *v*/*v*), and injected into the Agilent 1290 Infinity LCMS system.

### 4.4. Lipid Extraction from Plasma

Plasma samples were thawed on ice for pre-analytical processing. Ten microlitres of plasma were mixed with 180 μL chilled chloroform/methanol (1:2 *v*/*v*), vortexed for 15 s, and incubated at 4 °C for 1 h with agitation. Chilled chloroform (60 μL) and Milli-Q water (60 μL) were added, followed by vortexing and centrifugation (10,000 rpm, 7 min) to separate phases. The organic phase was collected and re-extracted with 500 μL chloroform. Combined extracts were reconstituted in chilled chloroform/methanol (1:1 *v*/*v*) for analysis.

### 4.5. Lipidomic Profiling

Lipid extracts were analysed using reverse-phase LCMS with an Agilent 1290 Infinity LC system and 6550 LC/ESI-Q-TOF MS in positive ionisation mode. LCMS analysis of the lipid extract was performed using an Agilent 1290 Infinity LC System on a C18 reverse-phase column (4.6 μm, 100 × 3.5 mm) with a 1290 Infinity UHPLC (0.5 μm Depth Filter × 0.004 in ID). The mobile phases consisted of (A) 0.1% formic acid and 10 mM ammonium acetate in MiliQ water, and (B) 0.1% formic acid, ten mM ammonium acetate in ACN/isopropanol (50/50, *v*/*v*) with flow rate of 0.6 mL/min. The column utilised a solvent gradient, and other parameters are detailed in Supplementary Table S2. Twenty microliters of each sample and 10 µL of SPLASH™ LIPIDOMIX^®^ internal standard were loaded into vials and placed in the auto-sampling tray. The column utilised a solvent gradient and an auto-sampler injected 2 μL from each vial, running a blank and quality control (QC) sample before sampling. Quality control (QC) samples, prepared by mixing equal amounts of plasma from all subjects, were used to control intra- and interbatch variability and injected every ten samples for each day.

### 4.6. Data Preprocessing and Annotation of Lipidomic Data

Raw MS data were converted to abf format and processed using MS-DIAL software 3.6 (RIKEN, Kanagawa, Japan) for feature detection, alignment, and metabolite identification based on the LipidBlast database. The MS1 and MS2 error ranges were set to 0.01 Da and 0.5 Da, respectively, with a 70% identification score cut-off. Lipids with fold changes > 10 and a coefficient of variation (CV%) < 30% in QC samples were analysed further. Normalisation was applied to correct biological and analytical variations [57].

### 4.7. Analysis of Lipidomic Data

Datasets containing *m*/*z* values, retention times, and peak areas were processed in MetaboAnalyst. Data underwent integrity checks, interquartile range filtering, log transformation, and auto-scaling. Univariate and multivariate analyses compared glioma grades (LGG, HGG) to controls. One-way ANOVA and FDR correction identified significant lipid metabolites (*p* < 0.05). Lipids with *p* < 0.05 and |log_2_FC| > 1 were considered differentially expressed. Partial least squares discriminant analysis (PLS-DA) and VIP scores ≥ 1 were used to identify critical lipids. Heatmaps visualised clustering and differential expression.

### 4.8. Lipid Biomarkers Screening

Biomarkers were screened using VIP scores (>1.5) and *p*-values (<0.05). ROC curves and AUC values assessed biomarkers’ diagnostic accuracy. AUC values of 0.75 or higher were considered to indicate acceptable predictive value. ROC analysis also evaluated combinations of significant lipids [58,59].

### 4.9. Correlation and Pathway Enrichment Analysis

Spearman’s correlation assessed relationships between plasma and tissue lipid metabolites. Pathway enrichment and topology analyses in MetaboAnalyst identified critical pathways, with impact scores estimating their relevance to glioma metabolism.

### 4.10. Statistical Analysis

Lipidomic data were analysed using SPSS 20.0. Results are presented as mean ± SD or percentages. For continuous variables, Student’s *t*-test (normally distributed) or Mann–Whitney U test (non-normally distributed) assessed differences between two groups, while one-way ANOVA with LSD was used for multigroup comparisons. Pearson’s χ^2^ test evaluated categorical variables. A *p*-value < 0.05 indicated statistical significance.

## 5. Conclusions

This study highlights that glioma pathogenesis significantly alters the lipidome in both tissue and plasma, with distinct lipid profiles and biomarker changes observed in glioma patients. Our findings show that lipidomics can differentiate glioma from non-gliomatous lesions and identify potential biomarker panels for diagnosis. The lipidome may also assist in monitoring metabolic changes and patient stratification. Furthermore, identification of plasma lipid contribute to the development of non-invasive approach for diagnosing and monitoring gliomas.

## Figures and Tables

**Table 1:** Clinical characteristics of glioma patients and control subjects.

Characteristics	Control (*n* = 11)	Low-Grade Glioma (*n* = 28)	High-Grade Glioma (*n* = 54)	*p*-Value
Gender—(*n*, %)				
Male	7 (63.6%)	11 (39.3%)	28 (51.9%)	0.512 ^a^, 0.283 ^b^
Female	4 (36.4%)	17 (60.7%)	26 (48.1%)	
Age—(years ± SD)				
Mean age	38.0 ± 13.763	37.6 ± 14.78	46.0 ± 15.66	**0.039 ^a^, 0.025 ^b^**
Range	21–59	19–68	20–78	
Ethnicity—(*n*, %)				
Malay	2 (18.2%)	9 (32.1%)	11 (20.4%)	0.400 ^a^, 0.245 ^b^
Chinese	8 (72.7%)	13 (46.4%)	26 (48.1%)	
Indian	1 (9.1%)	4 (14.3%)	15 (27.8%)	
Others	0 (0.0%)	2 (7.1%)	2 (3.7%)	
BMI—(kg/m^2^ ± SD)				
Mean BMI		23.2 ± 4.368	25.7 ± 4.076	**0.051 ^b^**
Range		16.5–30	19.2–33.9	
Clinical Manifestations—(*n*, %)				
Seizure	9 (81.8%)	23 (82.1%)	31 (57.4%)	**0.044 ^a^, 0.026 ^b^**
Headache	2 (18.2%)	7 (25.0%)	18 (33.3%)	0.146 ^a^, 0.274 ^b^
Body weakness	1 (9.1%)	4 (14.3%)	17 (31.5%)	0.179 ^a^, 0.189 ^b^
Speech disturbance	1 (9.1%)	4 (14.3%)	12 (22.2%)	0.480 ^a^, 0.393 ^b^
Memory impairment	0 (0.0%)	2 (7.1%)	7 (13.0%)	0.363 ^a^, 0.427 ^b^
Vomiting	2 (18.2%)	2 (7.1%)	7 (13.0%)	0.712 ^a^, 0.427 ^b^
Vision impairment	2 (18.2%)	2 (7.1%)	6 (11.1%)	0.846 ^a^, 0.568 ^b^
Reduced consciousness	0 (0.0%)	3 (10.7%)	2 (3.7%)	0.292 ^a^, 0.211 ^b^
Tumour location—(*n*, %)				
Frontal lobe	1 (9.1%)	10 (35.7%)	18 (33.3%)	0.185 ^a^, 0.393 ^b^
Temporal lobe	8 (72.7%)	10 (35.7%)	13 (24.1%)	
Parietal lobe	0 (0.0%)	2 (7.1%)	7 (13.0%)	
Occipital lobe	1 (9.1%)	0 (0.0%)	4 (7.4%)	
Temporal parietal lobe	0 (0.0%)	3 (10.7%)	8 (14.8%)	
Fronto-Temporal lobe		1 (3.6%)	3 (5.6%)	
Frontal Parietal	0 (0.0%)	1 (3.6%)	0 (0.0%)	
Brainstem		1 (3.6%)	0 (0.0%)	
Fronto-parieto-temporal lobe	1 (9.1%)	0 (0.0%)	1 (1.9%)	
Histological Diagnosis—(*n*, %)				
Control				
Mesial Temporal Sclerosis	4 (36.4%)			
Dysplasia	2 (18.2%)			
Epilepsy	2 (18.2%)			
Meningioma	1 (9.1%)			
Cavernoma	1 (9.1%)			
Tuberculoma	1 (9.1%)			
Glioma Grades and Subtypes—(*n*, %)				
Grade I		*n* = 5		
DNET		2 (7.1%)		
Ganglioma		1 (3.6%)		
Pilocytic astrocytoma		1 (3.6%)		
Glioneuronal tumour		1 (3.6%)		
Grade II		*n* = 23		
Oligodendroglioma		9 (32.1%)		
Diffuse astrocytoma		9 (32.1%)		
Gemistocytic astrocytoma		2 (7.1%)		
Pleomorphic xanthoastrocytoma		1 (3.6%)		
Pilocytic astrocytoma		1 (3.6%)		
Low-grade glioma		1 (3.6%)		
Grade III		*n* = 15		
Anaplastic astrocytoma		14 (21.7%)		
Anaplastic oligodendroglioma		1 (2.2%)		
Grade IV		*n* = 39		
Glioblastoma		38 (81.4%)		
Treatment protocol—(*n*, %)				
Surgery		21 (75.0%)	2 (3.7%)	
Surgery and adjuvant	11 (100%)	5 (17.9%)	46 (85.2%)	**<0.001 ^a,b^**
Surgery and Palliative Care		2 (7.1%)	6 (11.1%)	
Extent of surgery—(*n*, %)				
Excision		20 (71.4%)	37 (68.5%)	
Debulking		5 (17.9%)	15 (27.8%)	0.956 ^b^
Stereotactic Biopsy		3 (10.7%)	2 (3.7%)	
Patient’s Survival				
KPS (median) *		80	60	**<0.001 ^b^**
OS (months) *		25.9 ± 20.793	15.4 ± 14.868	**0.030 ^b^**
TTP (months) *		13.1 ± 10.660	3.35 ± 4.256	**<0.001 ^b^**
Number of deceased		4	5	0.949 ^b^

Data are presented as the mean ± SD. * Patient survivals were analysed using the Mann-Whitney U tests. Statistical significance was set at *p*-values < 0.05 and is depicted in bold. ^a^ Statistical tests were carried out between the control, LGG and HGG groups. ^b^ Statistical tests were performed between LGG and HGG groups. Abbreviations: BMI body mass index, DNET dysembryoplastic neuroepithelial tumour, HGG high-grade glioma, KPS Karnofsky Performance Scale, LGG low-grade glioma, *n* number of patients, OS overall survival, SD standard deviation, TTP time-to-progression.

**Table 2:** Potential lipid biomarkers based on univariate, multivariate and biomarker analysis in tissue samples of glioma patients.

Lipid Species	Lipid Category	RT (mins)	*m*/*z*	*^a^ p*-Value	*^b^* FDR	*^c^* Log_2_FC	*^d^* VIP Score	*^e^* AUC (95% CI)	*^f^* Glioma vs. Control
Tissue									
Cer 39:5	SP	4.97	598.53	3.23 × 10^−19^	8.67 × 10^−16^	3.06	3.97	0.986 (0.950–1.000)	Downregulated
LysoPC 21:3	GP	4.16	582.35	2.93 × 10^−7^	1.97 × 10^−4^	2.02	3.62	0.925 (0.853–0.989)	Downregulated
DG 43:11	GL	18.48	723.50	7.80 × 10^−6^	3.5 × 10^−3^	1.81	3.12	0.906 (0.819–0.976)	Downregulated
NAGlySer 14:0;O(FA 28:6)	FA	18.15	800.61	1.89 × 10^−5^	7.3 × 10^−3^	1.84	3.09	0.900 (0.812–0.972)	Downregulated
PC O-37:6	GP	0.86	778.58	9.57 × 10^−5^	0.021	2.88	4.54	0.831 (0.606–0.989)	Downregulated
Cer 49:12;4O	SP	14.58	758.57	0.000303	0.045	1.70	4.13	0.822 (0.674–0.944)	Downregulated
Plasma									
PE 6:0_15:3	GP	4.10	532.30	2.65 × 10^−5^	0.010	2.55	4.23	0.862 (0.719–0.961)	Downregulated
CerP 26:2;2O	SP	4.63	504.34	3.39 × 10^−6^	0.003	3.41	4.57	0.861 (0.735–0.958)	Downregulated
SM 24:3;2O	SP	4.65	559.39	9.62 × 10^−5^	0.021	−10.37	5.51	0.858 (0.788–0.918)	Upregulated
PC O-35:8	GP	1.06	746.51	5.88 × 10^−5^	0.016	2.48	4.82	0.856 (0.724–0.950)	Downregulated
DGGA 36:7	GL	12.02	804.52	1.39 × 10^−5^	0.009	3.03	5.85	0.844 (0.702–0.940)	Downregulated
PI-Cer 41:8;3O	SP	12.23	880.53	9.34 × 10^−6^	0.006	1.89	6.12	0.842 (0.730–0.937)	Downregulated
DG 22:1	GL	12.63	449.32	2.21 × 10^−5^	0.010	3.37	5.38	0.837 (0.699–0.956)	Downregulated
NAGlySer 9:0;O(FA 28:4)	FA	2.47	734.57	3.12 × 10^−5^	0.011	3.06	4.87	0.795 (0.646–0.927)	Downregulated
NAGlySer 13:0;O	FA	4.76	375.26	1.52 × 10^−4^	0.026	−3.98	1.17	0.788 (0.695–0.877)	Upregulated

*^a^ p*-values obtained from one-way ANOVA analysis. *^b^ p*-value adjusted for multiple comparisons based on FDR. *^c^* Fold change (FC) was obtained by comparing those lipid metabolites in the glioma with the control group; FC with a value > 1 indicates a relatively higher concentration present in the glioma compared to the control group. Fold change values greater than 1 indicate up-regulation, while values less than −1 indicate down-regulation. *^d^* Variable in Projection (VIP) was obtained from PLSDA with a threshold of 1.5. *^e^* Expression of lipid biomarkers in glioma as compared with control samples. *^f^* Lipid metabolites expression in glioma as compared to control Abbreviations: AUC area under the receiver operating characteristic curve (ROC), Cer Ceramide, DG Diacylglycerol, DGGA diacylglyceryl glucuronide, FDR false discovery rate, LPC lysophosphatidylcholine, *m*/*z* mass to charge ratio, mins minutes, NAGlySer N-acylglycine serine, PC-O Ether-linked phosphatidylcholine, RT retention time.

## Data Availability

The data supporting the findings of this study are available from the corresponding author upon reasonable request, subject to privacy and ethical restrictions.

## Supplementary Materials

The following supporting information can be downloaded at: https://www.mdpi.com/article/10.3390/ijms26199820/s1.

## Abbreviations

The following abbreviations are used in this manuscript:

AUC	Area under curve
BMI	Body mass index
Cer	Ceramide
CerP	Ceramide 1-phosphate
CNS	Central nervous system
DG	Diacylglycerol
DGGA	Diacylglyceryl glucuronide
DNET	Dysembryoplastic neuroepithelial tumour
FAS	Fatty acid synthase
FC	Fold Change
GDNF	Glial cell-derived neurotrophic factor
GPCR	G-protein-coupled receptor
HGG	High-grade glioma
KPS	Karnofsky Performance Scale
LC/ESI-Q-TOF MS	Liquid chromatography and electrospray ionisation quadrupole time-of-flight mass spectrometry
LCMS	Liquid chromatography-mass spectrometry
LGG	Low-grade glioma
LPC	Lysophosphocholine
MRI	Magnetic resonance imaging
NAGlySer	N-acylglycine serine
OS	Overall survival
PC O	Ether-linked phosphatidylcholine
PE	Phosphatidylethanolamine
PI-Cer	Ceramide phosphoinositol
PLSDA	Partial least squares-discriminant analysis
ROC	Receiver operating characteristic
RTK	Receptor tyrosine kinase
SD	Standard deviation
SM	Sphingomyelin
TTP	Time-to-progression
VEGF	Signalling, vascular endothelial growth factor
VIP	Variable in Projection
WHO	World Health Organization
