# Supplementary material for: Lipid Biomarkers in Glioma: Unveiling Molecular Heterogeneity Through Tissue and Plasma Profiling
Source: Int J Mol Sci. 2025 Oct 9;26(19):9820. doi: 10.3390/ijms26199820 (PMC12525097; doi:10.3390/ijms26199820)
Supplement: Supplementary file 1 [file ijms-26-09820-s001.zip › ijms-3852437-supplementary.pdf]

LIPID BIOMARKERS IN GLIOMA: UNVEILING MOLECULAR HETEROGENEITY THROUGH TISSUE AND PLASMA PROFILING

SUPPLEMENTARY

Supplementary Table S1: Differential lipid species in tissue and plasma of glioma patients compared to control.

No.	Lipid Compound	Class	*p*-value	FDR	F Value
		Tissue			
**1**	Cer 39:5;3O	Sphingolipid	3.23×10^{-19}	8.67×10^{-16}	112.26
**2**	PC O-33:5	Glycerophospholipid	1.30×10^{-12}	1.74×10^{-9}	49.72
**3**	NAGly 8:0;O(FA 24:2)	Fatty Acyl	1.39×10^{-9}	1.24×10^{-6}	31.53
**4**	LPC 21:3	Glycerophospholipid	2.93×10^{-7}	1.97×10^{-4}	20.63
**5**	PC 33:1	Glycerophospholipid	2.20×10^{-6}	1.18×10^{-3}	17.10
**6**	DG 43:11	Glycerolipid	7.80×10^{-6}	3.49×10^{-3}	15.02
**7**	NAGlySer 14:0;O(FA 28:6)	Fatty Acyl	1.89×10^{-5}	7.26×10^{-3}	13.62
**8**	PC 33:2	Glycerophospholipid	3.51×10^{-5}	0.01	12.68
**9**	DG 30:4	Glycerolipid	3.91×10^{-5}	0.01	12.52
**10**	TG 42:1	Glycerolipid	4.21×10^{-5}	0.01	12.41
**11**	PC O-31:2	Glycerophospholipid	7.76×10^{-5}	0.02	11.50
**12**	PC O-37:6	Glycerophospholipid	9.57×10^{-5}	0.02	11.20
**13**	CAR 20:4	Fatty Acyl	1.08×10^{-4}	0.02	11.02
**14**	TG 46:7	Glycerolipid	1.19×10^{-4}	0.02	10.89
**15**	PC O-33:6	Glycerophospholipid	1.32×10^{-4}	0.02	10.74
**16**	Cer 37:6;2O	Sphingolipid	1.69×10^{-4}	0.03	10.38
**17**	PC O-39:6	Glycerophospholipid	1.98×10^{-4}	0.03	10.16
**18**	Cer 49:12;4O	Sphingolipid	3.03×10^{-4}	0.05	9.57
		Plasma			
1	SHexCer 46:8;2O	Sphingolipid	5.69×10^{-10}	3.85×10^{-6}	28.01
2	VAE 20:3	Prenol lipid	6.83×10^{-9}	2.31×10^{-5}	23.93
3	PS 37:8	Glycerophospholipid	1.43×10^{-7}	3.22×10^{-4}	19.27
4	DGTS 36:7	Glycerolipid	2.54×10^{-7}	4.30×10^{-4}	18.42
5	Hex2Cer 40:7;2O	Sphingolipid	1.05×10^{-6}	1.42×10^{-3}	16.40
6	Hex2Cer 44:7;2O	Sphingolipid	1.35×10^{-6}	1.50×10^{-3}	16.04
7	DG 38:1	Glycerolipid	1.55×10^{-6}	1.50×10^{-3}	15.85
8	TG 52:12;4O	Glycerolipid	1.98×10^{-6}	1.68×10^{-3}	15.51
9	CerP 26:2;2O	Sphingolipid	3.39×10^{-6}	2.55×10^{-3}	14.77
10	PI-Cer 41:8;3O	Sphingolipid	9.34×10^{-6}	6.32×10^{-3}	13.41
11	Cer 12:2;3O/1:0;(2OH)	Sphingolipid	1.69×10^{-5}	9.08×10^{-3}	12.62
12	LPE 18:1(d7)	Glycerophospholipid	1.75×10^{-5}	9.08×10^{-3}	12.58
13	DG 22:1	Glycerolipid	2.21×10^{-5}	9.96×10^{-3}	12.27
14	NAGly 26:6;O(FA 28:6)	Fatty acyl	2.31×10^{-5}	9.96×10^{-3}	12.22
15	Cer 38:0;2O	Sphingolipid	2.56×10^{-5}	9.96×10^{-3}	12.08

16	SM 36:2;2O	Sphingolipid	2.58×10^{-5}	9.96×10^{-3}	12.07
17	PE 21:3	Glycerophospholipid	2.65×10^{-5}	9.96×10^{-3}	12.04
18	DG 24:3	Glycerolipid	2.88×10^{-5}	0.01	11.93
19	NAGlySer 9:0;O(FA 28:4)	Fatty acyl	3.12×10^{-5}	0.01	11.83
20	DG 53:9	Glycerolipid	3.42×10^{-5}	0.01	11.71
21	NAGlySer 16:3;O	Fatty acyl	3.56×10^{-5}	0.01	11.65
22	PS 27:4	Glycerophospholipid	5.39×10^{-5}	0.02	11.12
23	PC O-17:8	Glycerophospholipid	5.88×10^{-5}	0.02	11.01
24	LDGCC 12:0	Glycerolipid	5.99×10^{-5}	0.02	10.99
25	DGDG O-38:3	Glycerolipid	6.84×10^{-5}	0.02	10.82
26	PC O-35:5	Glycerophospholipid	7.73×10^{-5}	0.02	10.67
27	Cer 32:6;2O	Sphingolipid	8.18×10^{-5}	0.02	10.59
28	PC 21:3	Glycerophospholipid	8.84×10^{-5}	0.02	10.50
29	SM 24:3;2O	Sphingolipid	9.62×10^{-5}	0.02	10.39
30	SHexCer 34:4;3O/22:3;O	Sphingolipid	9.76×10^{-5}	0.02	10.37
31	LPE 20:5	Glycerophospholipid	1.12×10^{-4}	0.02	10.20
32	PC 21:2	Glycerophospholipid	1.17×10^{-4}	0.02	10.15
33	DGDG O-30:6	Glycerolipid	1.32×10^{-4}	0.03	9.99
34	DG 21:0	Glycerolipid	1.35×10^{-4}	0.03	9.96
35	HBMP 46:8	Glycerophospholipid	1.36×10^{-4}	0.03	9.95
36	CAR 18:2	Fatty acyl	1.43×10^{-4}	0.03	9.89
37	NAGlySer 13:0;O	Fatty acyl	1.52×10^{-4}	0.03	9.82
38	CerP 39:3;2O	Sphingolipid	1.53×10^{-4}	0.03	9.81
39	DGCC 25:3	Glycerolipid	1.59×10^{-4}	0.03	9.77
40	PS 42:4	Glycerophospholipid	1.59×10^{-4}	0.03	9.76
41	Cer 36:2;3O	Sphingolipid	1.74×10^{-4}	0.03	9.65
42	DG 25:3	Glycerolipid	1.83×10^{-4}	0.03	9.59
43	SQDG 43:11	Glycerolipid	2.07×10^{-4}	0.03	9.44
44	Cer 56:0;2O	Sphingolipid	2.24×10^{-4}	0.03	9.34
45	SHexCer 25:1;2O	Sphingolipid	2.70×10^{-4}	0.04	9.11
46	NAGlySer 17:2;O	Fatty acyl	2.94×10^{-4}	0.04	9.01

Abbreviations: CAR acylcarnitine, Cer Ceramide, CerP Ceramide 1-phosphate, DG Diacylglycerol, DGCC Diacylglycerol 3-O-carboxyhydroxymethylcholine, DGDG-O Ether-linked digalactosyldiacylglycerol, DGTS Diacylglyceryl trimethylhomoserine, FDR false discovery rate, HBMP Hemibismonoacylglycerophosphate, Hex2Cer Dihexosylceramide, LDGCC Lysodiacylglyceryl-3-O-carboxy hydroxymethyl choline, LPC lysophosphatidylcholine, LPE Lysophosphatidylethanolamine, NAGly N-acylglycine, NAGlySer N-acylglycine serine, PC Phosphatidylcholine, PC-O Ether-linked phosphatidylcholine, PE Phosphatidylethanolamine, PI-Cer Ceramide phosphoinositol, PS Phosphatidylserine, SHexCer Sulfatide, SQDG Sulfoquinovosyl diacylglycerol, SM Sphingomyelin, TG Triacylglycerol, VAE Vitamin A fatty acid ester.

Supplementary Figure S1: Differential characteristics of the lipid profile panel, composed of six lipids, were used to distinguish glioma from non-glioma tissue.

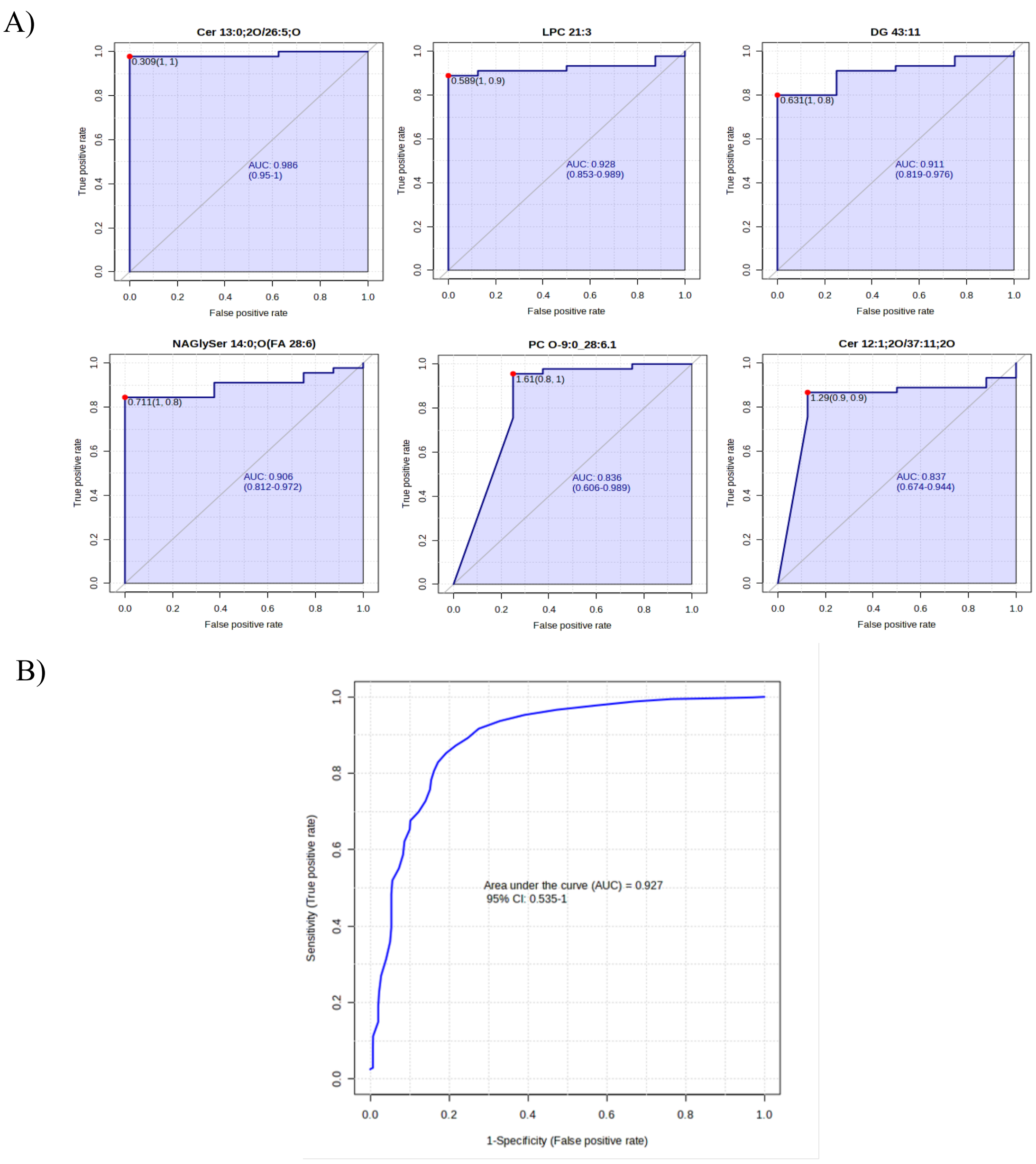

Abbreviations: AUC area under the receiver operating characteristic curve (ROC), Cer Ceramide, DG Diacylglycerol, LPC lysophosphatidylcholine, NAGlySer N-acylglycine serine, PC-O Ether-linked phosphatidylcholine.

Supplementary Figure S2: Differential characteristics of the lipid profile panel, composed of ten lipids, were used to distinguish glioma from non-glioma serum.

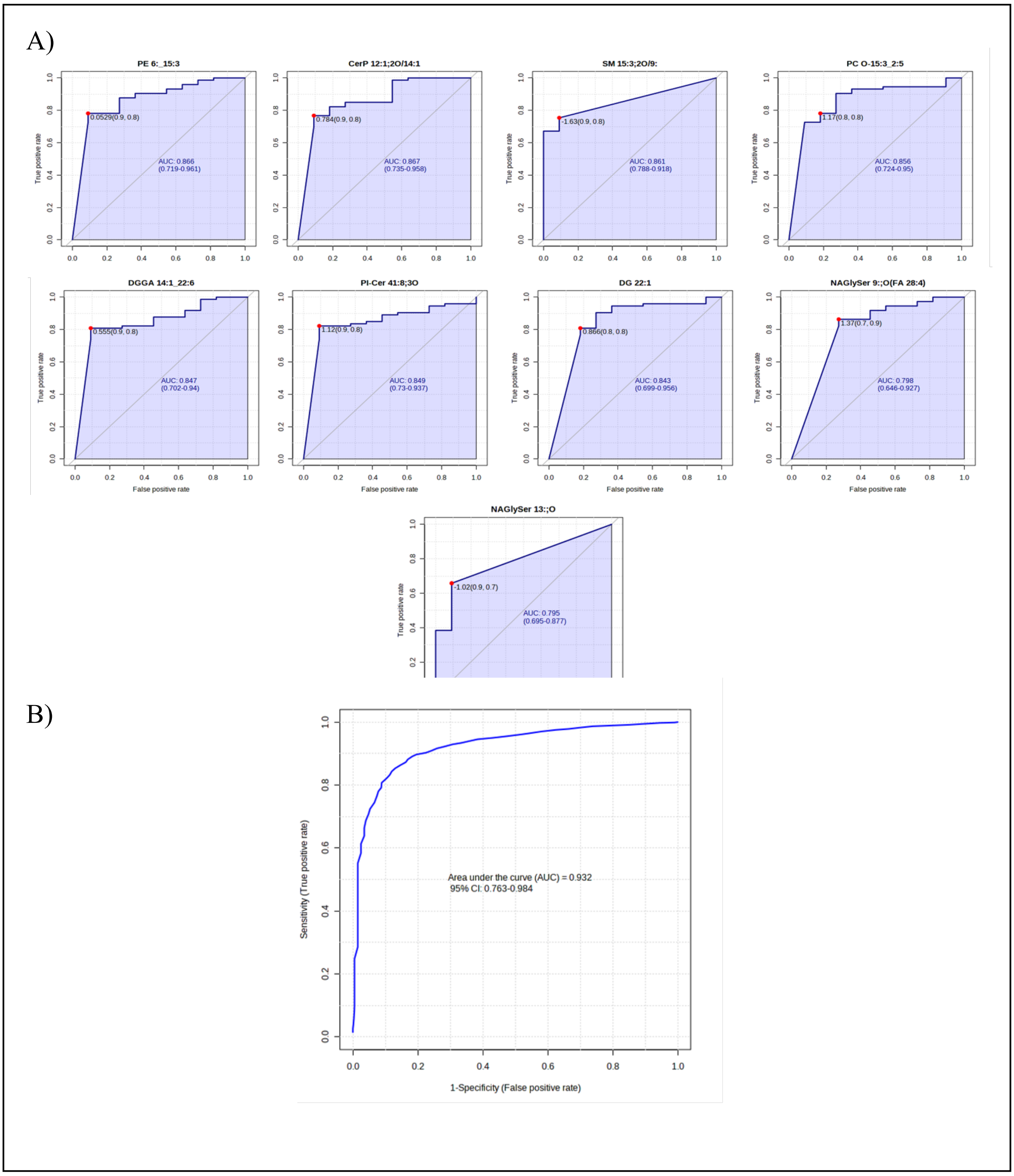

Abbreviations: AUC area under the receiver operating characteristic curve (ROC), CerP Ceramide 1-phosphate, DG Diacylglycerol, DGGA Diacylglyceryl glucuronide, NAGlySer N-acylglycine serine, PC-O Ether-linked phosphatidylcholine, PE Phosphatidylethanolamine, PI-Cer Ceramide phosphoinositol, SM Sphingomyelin.

Supplementary Table S2: Chromatographic conditions for positive mode ionisation.

**Parameter**	**Agilent 1290 Infinity II LC**
Analytical column	Agilent InfinityLab Poroshell 120 EC-C18, 3.0 x 100 mm, 2.7 µm (p/n 695975-302)
Guard column	Agilent InfinityLab Poroshell 120 EC-C18, 3.0 x 5 mm, 2.7 µm (p/n 823750-911)
Column temperature	50 °C
Injection volume	2 µl
Autosampler temperature	50 °C
Needle wash	15 seconds in wash port (50:50 methanol/isopropanol)
Mobile phase	A. 10 mM ammonium acetate, 0.2 mM ammonium fluoride in 9:1 water/methanol<br>B. 10 mM ammonium acetate, 0.2 mM ammonium fluoride in 2:3:5 acetonitrile/methanol/isopropanol
Flow rate	0.6 mL/min
Gradient program	Time (min) %B<br>0.00 70<br>1.00 70<br>3.50 86<br>10.00 86<br>11.00 100<br>17.00 100<br>17.10 70<br>19.00 70
Stop time	19 minutes
Post time	None
Observed column pressure	170 to 330 bar
